# Child characteristics and early intervention referral and receipt of services: a retrospective cohort study

**DOI:** 10.1186/s12887-020-1965-x

**Published:** 2020-02-22

**Authors:** Beth M. McManus, Zachary Richardson, Margaret Schenkman, Natalie J. Murphy, Rachel M. Everhart, Simon Hambidge, Elaine Morrato

**Affiliations:** 1 Department of Health Systems, Management and Policy, Colorado School of Public Health, 13001 E 17th Place, MS B119, Aurora, Colorado 80045 USA; 2 Physical Therapy Program, University of Colorado School of Medicine, 13121 East 17th Ave. Mail Stop C244, Aurora, Colorado 80045 USA; 3 Ambulatory Care Services Data and Analytics Denver Health, 777 Bannock St., Denver, Colorado 80204 USA; 4 Denver Community Health Services, 777 Bannock St., Denver, Colorado 80204 USA

**Keywords:** Early intervention, Developmental disabilities, Developmental delays, Safety net population, Health services research, Linked databases

## Abstract

**Background:**

Early Intervention (EI) is a federally mandated, state-administered system of care for children with developmental delays and disabilities under the age of three. Gaps exist in the process of accessing EI through pediatric primary care, and low rates of EI access are well documented and disproportionately affect poor and minority children. The aims of this paper are to examine child characteristics associated with gaps in EI (1) referral, (2) access and (3) service use. To our knowledge, this is the first study to leverage linked safety net health system pediatric primary care and EI records data to follow EI-referred children longitudinally to understand EI service use gaps from EI referral to EI service utilization.

**Methods:**

In a retrospective cohort design (14,710 children with developmental disability or delay), we linked pediatric primary care records between a large, integrated safety net health system in metro Denver and its corresponding EI program (2014–2016). Using adjusted marginal effects [ME, (95% CI)], we estimated gaps in EI referral, access, and service type (i.e., physical [PT], occupational [OT], speech therapy [ST] and developmental intervention [DI]). Analyses accounted for child characteristics including socio-demographics, diagnosis, condition severity, and baseline function.

**Results:**

Only 18.7% of EI-eligible children (*N* = 2726) received a referral; 26% of those (*N* = 722) received services for a net enrollment rate of 5% among EI-eligible children. Having the most severe developmental condition was positively associated with EI referral [ME = 0.334 [0.249, 0.420]) and Individualized Family Services Plan (IFSP) receipt [ME = 0.156 [0.088, 0.223]). Children less likely to be EI-referred were Black, non-Hispanic (BNH) [ME = -0.029 (− 0.054, − 0.004)] and had a diagnosed condition ([ME = − 0.046 (− 0.087, − 0.005)]. Children with a diagnosis and those with higher income were more likely to receive PT or OT. Higher baseline cognitive and adaptive skills were associated with lower likelihood of PT [ME = -0.029 (− 0.054, − 0.004)], OT [ME = -0.029 (− 0.054, − 0.004)], and ST [ME = -0.029 (− 0.054, − 0.004)].

**Conclusions:**

We identified and characterized gaps in EI referral, access, and service use in an urban safety-net population of children with high rates of developmental delay. Interventions are needed to improve integrated systems of care affecting primary care and EI processes and coordination.

## Background

Part C of the Individuals with Disabilities Education Act authorizes states to establish statewide early intervention (EI) systems for infants and toddlers with developmental delays and diagnosed conditions [1]. Enrollment in EI is a multi-step process involving (1) screening and identification of eligible children, (2) referral of eligible children to EI services, (3) evaluation and determination of eligibility, and (4) access to EI services, which includes receipt of an EI care plan, as well as receiving billable services from an EI program. The core EI services consist of physical [PT], occupational [OT], speech [ST] therapy, and developmental intervention [DI]. Low rates of EI access have been well documented and disproportionately affect poor and minority children [2, 3]. The benefits of early developmental interventions have been described, [4, 5] but quantifying consequences of EI access gaps remains challenging. To mitigate EI access gaps, the American Academy of Pediatrics created an algorithm to improve identification and referral of EI-eligible children [6]. Some systematic developmental screening initiatives have been partially successful and have improved EI referral rates, yet, in general, low EI referral rates persist [7–11]. There is evidence that referrals are missed due to simple oversight or communication failure between primary care, EI, and families as referral outcomes often are not formally tracked, and many EI-eligible children are not receiving EI services [12, 13]. Since gaps in referral have been identified but are not yet well characterized, the first aim of this paper is to examine child characteristics associated with EI referral gaps.

Historically, research and interventions tend to focus on the primary care system and gaps between EI referral and receipt of EI eligibility evaluation, [8–11, 14] rather than following children longitudinally and focusing on long-term systems coordination. Achieving actual receipt of services entails joint responsibility of primary care, EI, and families. Pediatric providers guide and encourage EI engagement, EI programs facilitate care, while families must agree to treatment and engage in services. However, current models of primary care-EI coordination are apparently insufficient in tracking EI-referred patients [15, 16]. Thus, Aim 2 is to examine child and family characteristics of EI-referred patients in relation to ultimately receiving an EI care plan, known as an Individualized Family Services Plan (IFSP), as a measure of receipt of services. We additionally follow the process one step further, as clinically determining need for EI-therapy is challenging and relying on diagnostic categories alone is insufficient [17]. Therefore, Aim 3 of this study is to examine child and family characteristics by EI service type(s) received as well as number of services received.

To our knowledge, this is the first study to leverage linked safety net health system pediatric primary care and EI records data to follow very low-income, predominantly minority EI-referred children longitudinally to understand EI service use gaps from EI referral to IFSP receipt to EI services receipt.

## Methods and declarations

### Study design and population

This was a retrospective cohort study linking primary care electronic health record data from Denver Health, a large, integrated safety net health system in Metro Denver, and administrative data from Rocky Mountain Human Services Early Intervention Program (RMHS) EI records. The cohort included children with developmental delay or disability who received at least one Denver Health well-child visit between 10/1/2014–6/30/2016. Denver Health includes a 477-bed hospital and 8 Federally Qualified health centers serving approximately 50,000 low-income children annually (35% of Denver children). Their database includes children’s clinical information, EI referral status, and sociodemographics. Previous research utilizing this database described service delivery gaps in pediatric obesity, [18] vaccines, [19] and developmental screening [20].

RMHS, located in Metro Denver, serves approximately 1000 families annually, including all Denver Health-referred patients. The RMHS database includes service use and functional outcomes. All children referred to RMHS between 10/1/14–9/30/16 (i.e., a three-month lag from Denver Health data collection) were included, allowing us to include at least a 3-month time lag between EI referral and EI access to account for the federal mandate requiring an IFSP is written within 45 days of EI referral.

The cohort included children less than 35 months of age with a diagnosed condition or developmental delay (described in Explanatory variables). Children were linked via first and last names and dates of birth across the Denver Health and EI databases using the reclink2 algorithm in Stata [21]. Observations were kept as acceptable matches if the resultant probability of an exact match was 85% or greater [22].

### Ethics approval and consent to participate

The Colorado Multiple Institutional Review Board approved this study and the Institutional Review Board at the Massachusetts Department of Public Health approved this study and the investigators’ access to the data.

### EI care variables

The primary dependent variables were EI referral (Aim 1), IFSP receipt (Aim 2), and EI service type and number of services used (Aim 3). *EI referral* was ascertained from the Denver Health record. At Denver Health, EI referrals are entered by a provider, electronically documented, and sent directly to RMHS through a HIPAA-compliant portal. *IFSP receipt* was collected from the RMHS EI database. *EI service type* was assessed from the RMHS EI data based on receipt of any PT, OT, ST, or DI. Total *number of EI services received* from EI was categorized as 1, 2, and 3 or more. Of note, the vast majority of EI services occur in the child’s home.

### Explanatory variables

*Covariates Ascertained from Denver Health*. Child’s race and ethnicity was categorized as white, non-Hispanic (WNH); black, non-Hispanic (BNH); Hispanic; and other race, non-Hispanic (ONH; includes Asian, Pacific Islander and more than one race). Annual household income (less than $20,000 or $20,000 or more) reflects the Federal Poverty threshold for a family of three [23]. We included infant birthweight (less than 1500 g, 1500–2500 g, and greater than 2500 g).

We also included measures of child’s condition type and severity. Data was pulled from Denver Health based on children having the required ICD-9 codes for the study, which we then split into two condition types: diagnosed condition and developmental delay. ICD-9 codes corresponding to diagnoses in the EI-Colorado Established Condition Database indicated a diagnosed condition. Common diagnosed conditions included Down syndrome, autism, cerebral palsy, and extremely low birth weight [24]. Developmental delay was indicated by ICD-9 code 315 (generalized developmental delay) without a co-occurring diagnosed condition, which is typically attributed to children with a failed developmental screening. In Colorado, children are eligible for EI if they show a 25% delay in one or more developmental areas [25]. Of note, in 2012 Denver Health’s information technology system underwent a major transformation allowing utilization of patient health records to provide tailored care, staff resources, and care-management services [26]. Simultaneously, electronic EI referrals for presumably EI-eligible children were implemented. Thus, ascertainment of condition type (i.e., diagnosis or delay) and EI referral are based upon validated processes.

Severity was categorized according to a clinically actionable 4-Tier severity algorithm employed by Denver Health based on published predictive models and front-line clinical judgement [26]. The four Tiers are comprised of (1) no chronic condition and age appropriate health service utilization; (2) mild condition requiring minimally elevated health service utilization; (3) moderate condition necessitating moderately elevated health service; and (4) severe chronic condition requiring high health service utilization. Due to clinical presentation heterogeneity, condition type and severity sub-groups do not overlap perfectly, however, the majority of Tier 1 and many Tier 2 children had a developmental delay only whereas the large majority of children in Tiers 3 and 4 had a diagnosed condition (such as cerebral palsy or Down syndrome).

*Covariates Ascertained from RMHS*. We included measures of child’s sex (male/female), primary language spoken at home (English vs a language other than English), insurance type (private or public [includes Medicaid and Children’s Health Insurance Program]) and age at EI entry (less than 12 months, 12–24 months, or 25–35 months). EI providers collect functional outcomes data at EI entry and exit for mandated reporting to the Office of Special Education Programs [27] in the form of Child Outcomes Summary (COS) scores. COS has been shown to have high clinical utility [28–30] and measures children’s function in 1) Social-Emotional, 2) Acquiring and Using Knowledge and Skills (cognition), and 3) Adaptive/Self-Care skills. Each domain score is a composite of parent and provider report and formal assessment comparing the child’s function to that of typically developing peers on a 7-point scale [1=“very early skills” (i.e., child does not use any immediate foundational skills related to this outcome) to 7=“all skills expected” (i.e., no concerns about the child’s function)] [31]. More specifically, EI-CO developed age-anchored developmental tasks [32] to address each COS domain. Information to assess the acquisition of these developmental tasks can be ascertained from a combination of parent report, functional assessment results (e.g., EI-CO uses the Batelle Developmental Inventory-2 [BDI-2]), and provider clinical judgement. All CO-EI providers receive extensive training in the administration, scoring, an interpretation of BDI-2 and COS scores [33]. Yet, currently, RMHS does not have mechanisms in place to ensure routine COS collection, therefore there is not 100% compliance.

### Data analysis

We first calculated descriptive statistics for each variable. To describe associations between child characteristics and EI care variables, we fit a series of adjusted logit regressions to estimate EI referral for Aim 1, and IFSP receipt and service type for Aims 2 and 3. Adjusted models included child’s condition type, birthweight, age at EI entry, family income, race, ethnicity, sex, and baseline function. Analyses including baseline function were restricted to children with complete service use and COS information (*n* = 448).

To describe the association between child characteristics and the number of EI services received in Aim 3, we estimated adjusted ordinal logit regression models due to the ordered nature of service use categories. We estimated the marginal effects and 95% CI of receiving each service use category (i.e., relative to the others) for each child characteristic. Marginal effects are interpreted as probabilities and estimate the associations between child characteristics and service use category. Compared to odds ratios, they are more intuitive and are not affected by extremely common or rare events [34, 35]. All analyses were conducted in Stata v14.2 [21].

## Results

### Characteristics of study cohort

**Fig. 1:**
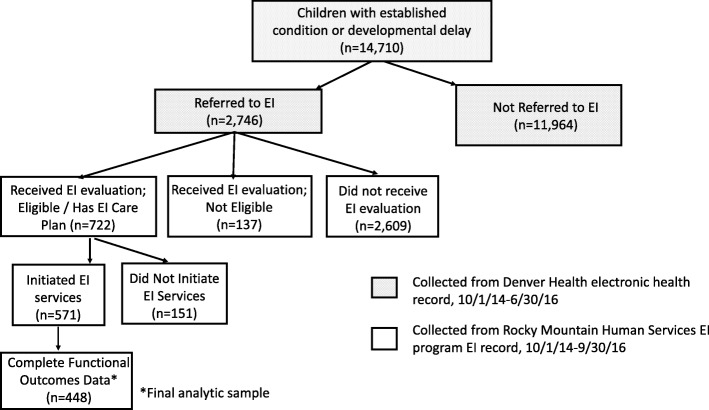
Consort Diagram of Study Sample Selection

The cohort included 14,710 children with either a developmental delay or a developmental disability. Of the 14,710 children, 2746 (18.7%) were referred to EI and of those referred, 722 (26.3%) received an IFSP (Fig. 1, Table 1). Of those with an IFSP, 571 initiated EI services, and of those, 448 had complete COS information (Fig. 1, Table 2). Children with and without complete COS information did not differ by clinical or sociodemographic characteristics (results not shown). Among children EI-referred and with an IFSP, 12.3% had a diagnosed condition, over 90% had an annual family income of less than $20,000, 73.7% were Hispanic, 15% were white, non-Hispanic (WNH), and 13% were black, non-Hispanic (BNH); 62.0% were under 12 months of age, and 70.6% had no chronic condition (*n* = 1).

**Table 1:** Characteristics of study sample children with developmental delays and disabilities (*n* = 14,710) within the study healthcare system

	Not Referred (*n* = 11,964)	Referred, but not Receiving EI (*n* = 2024)	Referred and Received IFSP (*n* = 722)
Condition Type			
Developmental Delay (DD)	97.5	90.5	87.7
Diagnosed Condition (DC)	2.5	9.5	12.3
Condition Severity Group			
No Condition	89.5	74.8	70.6
Mild	6.8	10.9	10.2
Moderate	2.0	9.0	11.0
Severe	1.6	5.4	8.2
Birthweight by Category			
Less than 1.5 kg	0.5	3.6	3.46
1.5 kg to 2.5 kg	4.8	8.3	8.45
Greater than 2.5 kg	94.7	88.1	88.1
Child’s Age			
Less than 12-Months	59.4	58.9	62.0
12–24 Months	21.0	23.3	28.7
Greater than 24 Months	19.6	17.8	9.3
Family Income (annually)			
Less than $19,999	93.8	94.6	91.8
Greater than $20,000	6.2	5.4	8.2
Race/Ethnicity			
White, non-Hispanic	15.0	14.4	14.5
Black, non-Hispanic	12.8	11.2	9.3
Hispanic	69.0	70.4	73.7
Other, non-Hispanic	3.2	4.0	2.5
Primary Language^A^			
English	–	–	55.0
Other	–	–	45.0
Child’s sex, Female	51.0	37.8	36.7
Insurance Type, Medicaid and CHP + ^A^	–	–	87.7

^A^Characteristics only collected through EI program, not pediatric primary care office

**Table 2:** Service Use among sample children who received EI referral and initiated EI services

Characteristic	% (n)
*Type of EI Services Received (n = 571)*^*A*^	
Physical Therapy	21.4 (122)
Occupational Therapy	17.3 (99)
Speech and Language Pathology	74.4 (425)
Developmental Intervention	49.4 (282)
*Number of EI Services Received (n = 571)*	
One Service	18.2 (104)
Two Services	35.2 (201)
Three or more Services	46.6 (266)
	Mean (SD)
Child Outcome Survey (COS) score at EI entry (*n* = 448)^B^	
Positive Social Emotional Skills	4.6 (1.8)
Acquiring and Using Knowledge and Skills	3.7 (1.5)
Taking Appropriate Action to Meet Needs	4.3 (1.7)

^A^Does not add up to 100% since some children received more than one type of EI service; sub-sample of study children who initiated EI services

^B^Each COS sub-scale score is derived from provider clinical judgment, parent concerns, and developmental assessment results and measured on a 7-point scale, from 1 = very early skills (i.e., child does not use any immediate foundational skills related to this outcome) to 7 = all skills expected (i.e., there are no concerns about the child’s function in this area)

Mean (SD) entry COS scores were 3.7 (1.5) for Acquiring and Using Knowledge and Skills, 4.3 (1.7) for Adaptive/Self-Care, and 4.6 (1.8) for Positive Social Emotional function. Twenty-one percent of the EI sample received PT, 17.3% received OT, 74.4% received ST, and 49.4% received DI (Table 2).

### Aim 1. Characteristics of EI-referral gaps

Children who had a diagnosis versus a developmental delay were 4.6% less likely (ME = -0.046 [− 0.087, − 0.005)] to be EI-referred. Normal birthweight (> 2500 g), and low birthweight (1501–2500 g) infants were 26.9% (ME = -0.269 [− 0.365, − 0.174) and 17.6% less likely (ME = -0.176 [− 0.276, − 0.077]) than very low birthweight (< 1500 g) infants to be referred. One-year olds were more likely to be EI-referred [ME = 0.023 [0.006, 0.041]) and 2-year olds were less likely to be EI-referred [ME = − 0.042 [− 0.058, − 0.025]) than infants. BNH children were 2.9% less likely than WNH children (ME = -0.029 [− 0.054, − 0.004]) to be referred. Children with severe, moderate, and mild conditions were 33.4% (ME = .334 [0.249, 0.420]), 33.8% (ME = 0.338 [0.314, 0.461]), and 9.8% (ME = 0.098 [0.070, 0.127]), respectively, more likely than children with no special health care condition to be referred (Table 3).

**Table 3:** Adjusted marginal effects [95% CI] of EI Referred Only (*n* = 2024) and Referred and Received IFSP (*n* = 722) sub-groups for each child characteristic

*Independent Variables*	Referred Only	Referred and Received IFSP
Diagnosed Condition^A^	-0.046*	−0.015
	[− 0.087, − 0.005]	[− 0.030, 0.001]
Condition Severity Group		
No Condition	*ref*	*ref*
Mild	0.098***	0.024**
	[0.070, 0.127]	[0.008, 0.040]
Moderate	0.388***	0.154***
	[0.314, 0.461]	[0.095, 0.213]
Severe	0.334***	0.156***
	[0.249, 0.420]	[0.088, 0.223]
Birthweight by Category		
Less than 1.5 kg	*ref*	*ref*
1.5 kg to 2.5 kg	−0.176***	− 0.000
	[−0.276, − 0.077]	[− 0.036, 0.036]
Greater than 2.5 kg	− 0.269***	− 0.021
	[− 0.365, − 0.174]	[− 0.054, 0.012]
Child’s Age		
Less than 12-Months	*ref*	*ref*
12–24 Months	0.023**	0.014**
	[0.006, 0.041]	[0.004, 0.025]
Greater than 24-Months	−0.042***	−0.031***
	[−0.058, − 0.025]	[− 0.039, − 0.023]
Family Income (annually)		
Greater than $20,000	− 0.009	0.011
	[−0.034, 0.017]	[−0.004, 0.027]
Race/Ethnicity		
White, non-Hispanic	*ref*	*ref*
Black, non-Hispanic	−0.029*	−0.012
	[−0.054, − 0.004]	[− 0.025, 0.002]
Hispanic	0.017	0.006
	[−0.002, 0.037]	[−0.005, 0.017]
Other, non-Hispanic	0.019	−0.015
	[−0.024, 0.061]	[−0.035, 0.004]
Child’s sex, Female	−0.078***	− 0.020***
	[−0.091, − 0.065]	[− 0.027, − 0.012]

^A^Diagnosed conditions for EI eligibility can be found in the Colorado Established Condition Database^20^, the referent group is children with developmental delay only

*p < .05; **p < .01; ***p < .001

### Aim 2. Characteristics of EI access gaps

Children with severe, moderate, and mild conditions were 15.6% (ME = 0.156 [0.088, 0.223]), 15.4% (ME = 0.154 [0.095, 0.213]), and 2.4% (ME = 0.024, [0.008, 0.040]), respectively, more likely than children with no special health care condition to receive an IFSP. One-year olds were more likely [ME = 0.014 [0.004, 0.025]) and two-year olds were less likely to receive an IFSP [ME = − 0.031 [− 0.039, − 0.023]) than infants (Table 3).

### Aim 3. Characteristics of EI service type gaps

Children with diagnosed conditions were 22.5% more likely to receive PT (ME = 0.225 [0.104, 0.347]), 21.4% more likely to receive OT (ME = 0.214 [0.091, 0.337]), and 22.4% more likely to receive DI (ME = 0.224 [0.088, 0.360]) than children with a developmental delay (Table 4), and were also more likely to receive three or more services (ME = 0.319 [0.199, 0.440]; Table 5). Low-income children were 13.6% less likely to receive PT (ME = -0.136 [− 0.201, − 0.071]) and 10.4% less likely to receive OT (ME = -0.104 [− 0.197, − 0.011]) than higher income children. Compared to children with birthweights less than 1500 g, children in other birthweight groups were more likely to receive PT. One-year olds were 11.4% less likely (ME = -0.114 [− 0.213, − 0.016]) and 2-year olds were 23.4% (ME = -0.234 [− 0.400, − 0.086]) less likely to receive DI than infants (Table 4).

**Table 4:** Adjusted marginal effects [95% CI] of receiving a core EI service among sample children with complete outcomes information^A^

*Independent Variables*	Marginal Effects of any PT	Marginal Effects of any OT	Marginal Effects of any ST	Marginal Effects of any DI
Had a Diagnosis	0.225***	0.214***	0.084	0.224**
	[0.104, 0.347]	[0.091, 0.337]	[−0.030, 0.197]	[0.088, 0.360]
Birthweight by Category				
Less than 1.5 kg	*ref*	*ref*	*ref*	*ref*
1.5 kg to 2.5 kg	0.177*	−0.056	−0.210	− 0.010
	[0.026, 0.328]	[−0.260, 0.148]	[−0.463, 0.044]	[− 0.331, 0.311]
Greater than 2.5 kg	0.135**	− 0.014	−0.102	− 0.121
	[0.041, 0.228]	[−0.194, 0.167]	[− 0.318, 0.115]	[− 0.406, 0.164]
Child’s Age				
Less than 12-Months	*ref*	*ref*	*ref*	*ref*
12–24 Months	− 0.024	−0.037	− 0.080	− 0.114*
	[− 0.096, 0.048]	[− 0.111, 0.036]	[− 0.179, 0.018]	[− 0.213, − 0.016]
Greater than 24-Months	0.013	0.045	0.034	− 0.234**
	[−0.112, 0.138]	[− 0.103, 0.192]	[− 0.103, 0.170]	[− 0.400, − 0.068]
Family Income (annually)				
Greater than $20,000	− 0.136***	− 0.104*	− 0.078	0.057
	[− 0.201, − 0.071]	[− 0.197, − 0.011]	[− 0.230, 0.075]	[− 0.092, 0.207]
Race/Ethnicity				
White, non-Hispanic	*ref*	*ref*	*ref*	*ref*
Black, non-Hispanic	0.034	−0.003	0.045	0.041
	[−0.125, 0.193]	[−0.157, 0.152]	[− 0.142, 0.232]	[− 0.156, 0.239]
Hispanic	0.001	−0.037	− 0.004	− 0.097
	[− 0.097, 0.099]	[− 0.144, 0.071]	[− 0.126, 0.118]	[− 0.243, 0.048]
Other, non-Hispanic	0.056	− 0.093	− 0.036	− 0.047
	[− 0.141, 0.253]	[− 0.357, 0.172]	[− 0.339, 0.267]	[− 0.332, 0.238]
Child’s sex, Female	0.028	− 0.054	− 0.026	− 0.006
	[− 0.040, 0.096]	[− 0.121, 0.014]	[− 0.112, 0.060]	[− 0.100, 0.087]
Primary Language, English	0.013	0.017	− 0.028	0.049
	[− 0.062, 0.088]	[− 0.062, 0.097]	[− 0.121, 0.064]	[− 0.053, 0.151]
Insurance Type, Medicaid and CHP+	− 0.067	0.007	0.039	0.157*
	[−0.186, 0.053]	[−0.109, 0.122]	[− 0.111, 0.188]	[0.005, 0.309]
Entry Child Outcome Summary Sub-Scales^B^				
Positive Social Emotional Skills	0.054***	−0.002	−0.017	− 0.073***
	[0.026, 0.082]	[−0.032, 0.028]	[−0.055, 0.020]	[− 0.112, − 0.034]
Acquiring and Using Knowledge and Skills	0.039**	0.009	−0.049*	0.032
	[0.010, 0.068]	[−0.021, 0.039]	[−0.086, − 0.011]	[−0.009, 0.072]
Taking Appropriate Action to Meet Needs	−0.095***	−0.051***	0.058***	0.033
	[−0.125, − 0.066]	[− 0.078, − 0.025]	[0.027, 0.090]	[−0.003, 0.068]

^A^Sample includes 448 children with complete baseline Child Outcomes Summary Data

*p < .05; **p < .01; ***p < .001

^B^Each COS sub-scale score is derived from provider clinical judgment, parent concerns, and developmental assessment results and measured on a 7-point scale, from 1 = very early skills (i.e., child does not use any immediate foundational skills related to this outcome) to 7 = all skills expected (i.e., there are no concerns about the child’s function in this area)

**Table 5:** Adjusted marginal effects [95% CI] of the number of EI services among sample children who initiated EI services and have complete child outcomes information^A^

	Marginal Effects of Number of Services (Compared to other Service Groups)		
*Independent Variables*	One Service	Two Services	Three or More Services
Had a Diagnosis	−0.143***	− 0.176***	0.319***
	[−0.194, − 0.092]	[− 0.257, − 0.095]	[0.199, 0.440]
Birthweight by Category			
Less than 1.5 kg	*ref*	*ref*	*ref*
1.5 kg to 2.5 kg	−0.025	− 0.018	0.043
	[−0.201, 0.151]	[−0.132, 0.096]	[− 0.247, 0.332]
Greater than 2.5 kg	0.001	0.000	−0.001
	[−0.161, 0.162]	[−0.099, 0.099]	[− 0.262, 0.260]
Child’s Age			
Less than 12-Months	*ref*	*ref*	*ref*
12–24 Months	0.078*	0.044*	−0.122*
	[0.006, 0.151]	[0.009, 0.078]	[−0.226, − 0.019]
Greater than 24-Months	0.073	0.042	−0.114
	[− 0.052, 0.197]	[−0.010, 0.093]	[− 0.288, 0.060]
Family Income (annually)			
Greater than $20,000	−0.054	− 0.044	0.098
	[−0.134, 0.025]	[−0.124, 0.036]	[− 0.060, 0.257]
Race/Ethnicity			
White, non-Hispanic	*ref*	*ref*	*ref*
Black, non-Hispanic	− 0.021	− 0.024	0.045
	[−0.111, 0.069]	[− 0.127, 0.079]	[− 0.148, 0.238]
Hispanic	0.061	0.046	−0.107
	[−0.015, 0.137]	[− 0.022, 0.114]	[− 0.249, 0.035]
Other, non-Hispanic	0.084	0.056	−0.140
	[−0.037, 0.205]	[−0.019, 0.132]	[− 0.331, 0.051]
Child’s sex, Female	− 0.003	− 0.002	0.005
	[−0.061, 0.054]	[− 0.039, 0.034]	[− 0.089, 0.100]
Primary Language, English	− 0.016	− 0.010	0.026
	[−0.079, 0.047]	[− 0.049, 0.029]	[− 0.075, 0.128]
Insurance Type, Medicaid and CHP+	− 0.040	− 0.021	0.061
	[−0.166, 0.086]	[− 0.074, 0.032]	[− 0.118, 0.239]
Child Outcomes Summary Sub-Scales^B^			
Positive Social Emotional Skills	0.027*	0.017*	−0.044*
	[0.002, 0.053]	[0.001, 0.033]	[−0.084, − 0.004]
Acquiring and Using Knowledge and Skills	−0.008	− 0.005	0.013
	[−0.032, 0.016]	[−0.020, 0.010]	[− 0.026, 0.052]
Taking Appropriate Action to Meet Needs	−0.007	− 0.004	0.012
	[−0.029, 0.014]	[−0.018, 0.009]	[− 0.023, 0.047]

^A^Sample includes 448 children with complete baseline Child Outcomes Summary Data

*p < .05; **p < .01; ***p < .001

^B^Each COS sub-scale score is derived from provider clinical judgment, parent concerns, and developmental assessment results and measured on a 7-point scale, from 1 = very early skills (i.e., child does not use any immediate foundational skills related to this outcome) to 7 = all skills expected (i.e., there are no concerns about the child’s function in this area)

Children with higher baseline Social-Emotional function were 5% more likely to receive PT (ME = 0.054 [0.026, 0.082]) and 7.3% less likely to receive DI (ME = -0.073 [− 0.112, − 0.034]. Children with higher baseline cognitive function were 3.9% more likely to receive PT (ME = 0.039 [0.010 0.068]) and 4.9% less likely to receive ST (ME = -0.049 [− 0.086, − 0.011]). Children with higher baseline Adaptive/Self-Care function were 5.1% less likely to receive OT (ME = -0.051 [− 0.078, − 0.025]), 5.1% less likely to receive ST (ME = -0.051 [− 0.078, − 0.025]), and 5.8% more likely to receive DI (ME = 0.058 [0.027, 0.090]) (Table 4).

## Discussion

Overall, we found significant gaps at each step of the EI referral-service delivery process. Among infants and toddlers with developmental delay or disability receiving care in a safety net health system, only 18.7% were EI-referred and only 26.3% of those EI-referred accessed EI (net access rate of less than 5% of all eligible children). Moreover, service type and breadth was associated with child’s income, diagnosis, and baseline function.

### EI referral gaps

A main finding of this study is that BNH children were less likely than their WNH counterparts to be EI-referred. While the reasons behind racial differences in EI referral are not explored in this study, these disparities in access have been reported [3]. A recent systematic review [36] of such research suggests that racial differences in care stem, in part, from differences in provider behavior. Providers tend to dismiss parental developmental concerns and abnormal developmental screening results and attribute them to social rather than clinical risk for BNH families compared to WNH, which could translate to fewer EI referrals.

The second main finding of this paper is that children with diagnosed conditions were less likely than their peers with a developmental delay to be EI-referred after accounting for condition severity. It may be that children with diagnosed conditions are being referred to specialty clinics more often and receiving care outside of EI [37]. In fact, one study found that half of birth to 3 year olds with a developmental condition receive clinic-based therapy services [38]. Additionally, children with more severe conditions are more likely to receive an EI referral regardless of their condition type. These findings highlight the clinical complexity of EI-eligible children and suggest that children most likely to receive an EI referral are those whose condition aligns with federal and CO-EI eligibility (e.g., developmental delay, extremely low birth weight, and high need special healthcare conditions).

### EI access gaps

The third main finding of this paper is that, compared to infants, 2-year olds are less likely to receive an IFSP. While this study cannot examine causality and there are limited comparable findings, it suggests caregivers of 2-year olds with developmental concerns need tailored support to navigate the EI system, especially since they typically interface less with primary care and have a shorter time window to access EI.

While we have limited comparisons for EI access findings, one study found that 30–50% of EI-referred children receiving care in urban pediatric practices received an EI evaluation, [11] fitting with 31% in our sample (Fig. 1). Moreover, we found that children with greater condition severity were more likely to access EI and that the majority of our cohort had a developmental delay (versus established condition), also consistent with previous literature [5, 36].

Reasons for access gaps may be numerous, and have been explored in previous studies [15, 16]. Future research should engage EI stakeholders (pediatricians, EI providers and parents) to understand EI enrollment barriers as previous research suggests that increasing family engagement [15] and implementing cross-system strategies such as centralized referrals and patient navigators can bolster EI enrollment [16].

### EI service type gaps

We found EI service use gaps among the poorest cohort children. In this safety net population, higher income may be a marker for greater economic resources, knowledge, or capacity to advocate for PT and OT and future research should explore EI therapy barriers and facilitators.

We also found that having a diagnosed condition (versus developmental delay) was associated with higher likelihood of receiving a greater breadth of EI services. While having a diagnosis seems to trigger an EI referral less often, once these children access EI they receive a greater breadth of services than their peers with developmental delays. Future research should focus on therapy use among EI-enrolled children as previous literature is mixed with regard to service type disparities [39].

This study also examined the association between routinely collected global measures of children’s function and EI service use. Baseline functional skills were associated with core EI service use in expected directions, suggesting baseline COS scores may be a good marker of EI therapy need. For example, children with higher social-emotional function likely have less need for DI, which focuses, in part, on promoting social skills.

### Limitations

The data were ascertained from one health system and EI program. However, Denver Health serves as medical home to 50,000 low-income children in Metro Denver and RMHS serves approximately 2/3 of Metropolitan Denver EI-eligible children. Thus, although results may not be generalizable to the general EI-population, they are applicable to lower-income, urban, EI-eligible children; and in fact, nearly 50% of EI-enrolled children nationally are low-income [5]. Related, the prevalence rate of children with developmental conditions in this sample (14,710/50,000 = 29.4%) is comparable to national estimates of low-income infants and toddlers with or at risk for developmental delays [40]. We were unable to examine reasons for EI referral (i.e., beyond a diagnosis), EI care quality, parental concerns, or capacity to navigate or advocate within the EI system. Previous research suggests that low EI enrollment rates can be explained, in part, by families who refuse services or do not follow up on EI referrals, [15] and families play an integral role in accessing EI services. Finally, we assume that children who received diagnostic code consistent with EI-eligible conditions in CO are, in fact, EI-eligible. However, we have no way to confirm this among the children who did not receive an EI evaluation. Yet, if pediatricians document a developmental delay in a child’s medical record, it seems plausible that developmental follow-up or referral is warranted. Additionally, Colorado eligibility for EI stipulates that a child has a 25% or greater delay in one or more developmental areas [25], and in this study we found that very few children who received an EI evaluation were found ineligible (Fig. 1). Future research should conduct qualitative interviews with providers and parents to understand care quality, including shared decision-making, and EI service gaps.

### Strengths

To our knowledge, this is the first study to link pediatric primary care and EI records to examine EI service use among a low-income EI-eligible cohort. We found differences in receipt of EI therapy services by condition type, income, and baseline function. These findings have important implications for EI providers, program directors, and policy makers for improving outreach and care planning. The methods of this study can serve as a model for leveraging electronic health data to examine EI service use gaps with an eye toward improving access, service use patterns, and functional outcomes.

Our findings of the prevalence of developmental delays is well aligned with national estimates of the prevalence of children at risk for developmental delay in low-income settings [40]. We also found that most children receiving EI services are doing so because of a developmental delay, rather than a diagnosed condition, which also matches previous findings [41] and bolsters the validity of our results. Moreover, our EI-referral rate of 18.7% is consistent with previous literature examining developmental follow-up for urban children with a failed developmental screening, [10] suggesting generalizability to similar populations.

## Conclusion and clinical implications

Our findings highlight the multiple decision-makers in the EI enrollment loop: pediatricians make an EI referral, EI providers recommend EI service type, and families make the final decisions regarding receipt of services. To minimize EI access gaps and ensure that all eligible children are referred to and receive EI services, our findings suggest opportunities for improved family engagement, in addition to communication and coordination between pediatric primary care and EI systems at each step of the EI enrollment process.

## Data Availability

The datasets generated and analyzed during the current study are not publicly available because they contain personal health information, but are available from the corresponding author on reasonable request.

## Abbreviations

CO: Colorado
COS: Child outcomes summary
DH: Denver Health
DI: Developmental intervention
EI: Early intervention
OT: Occupational therapy
PT: Physical therapy
RMHS: Rocky mountain human services
ST: Speech therapy
